# Supplementary material for: Shortening injection matrix for serial crystallography
Source: Sci Rep. 2020 Jan 9;10:107. doi: 10.1038/s41598-019-56135-1 (PMC6952439; doi:10.1038/s41598-019-56135-1)
Supplement: Supplementary file 1 — Supplementary Data [file 41598_2019_56135_MOESM1_ESM.docx]

**Supplementary Data**

**Shortening injection matrix for serial crystallography**

Ki Hyun Nam^1,2,*^

^1^ Division of Biotechnology, Korea University, Seoul, Republic of Korea.

^2^ Institute of Life Science and Natural Resources, Korea University, Seoul, Republic of Korea.

*Corresponding author.

Division of Biotechnology, Korea University, Seoul, Republic of Korea. E-mail: structures@korea.ac.kr (K.H.N.)


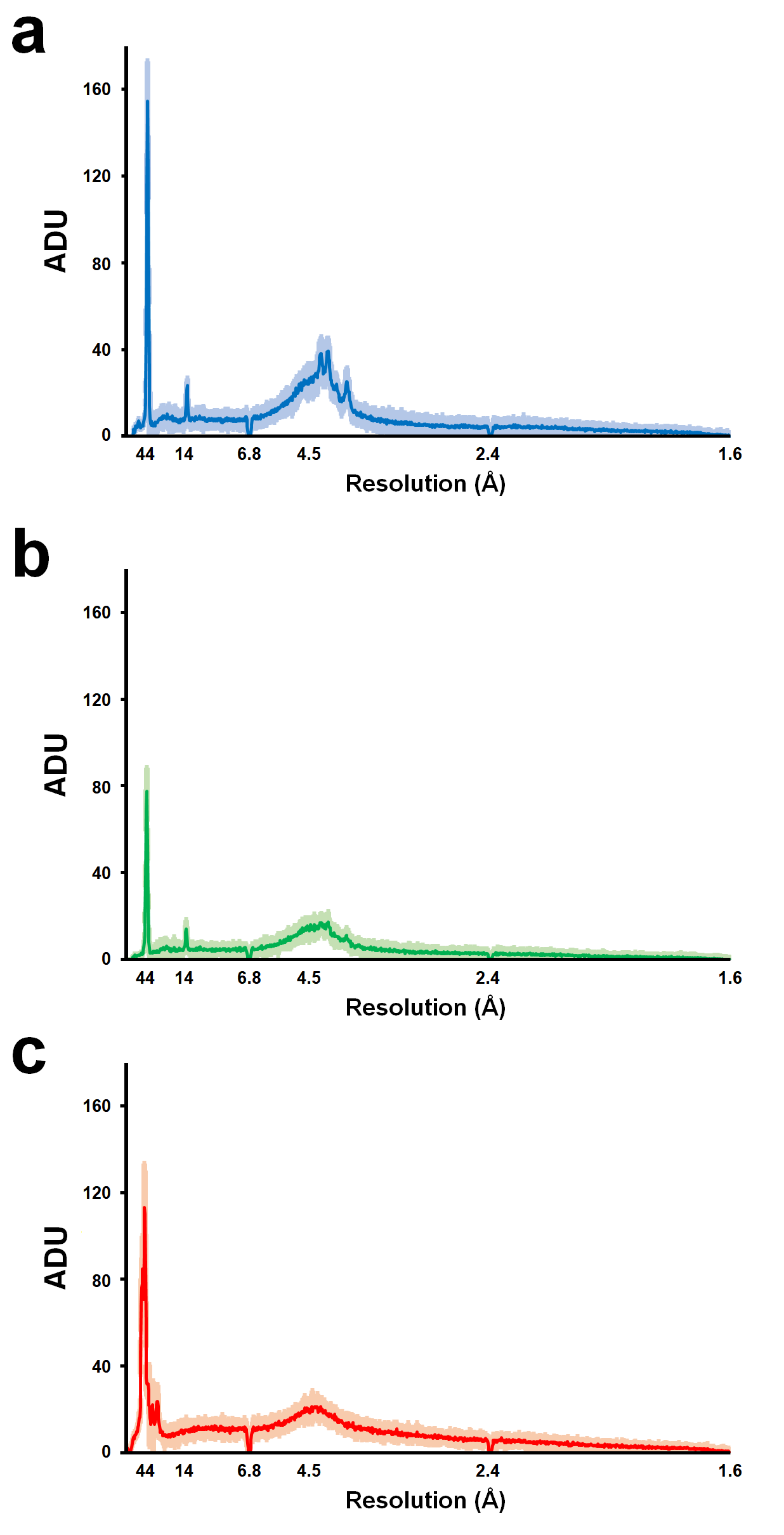


**Supplementary Figures S1.** Intensity of background scattering of (a) shortening A, (b) shortening B, and (c) LCP. The average intensity and standard deviation are indicated by line and transparent regions.


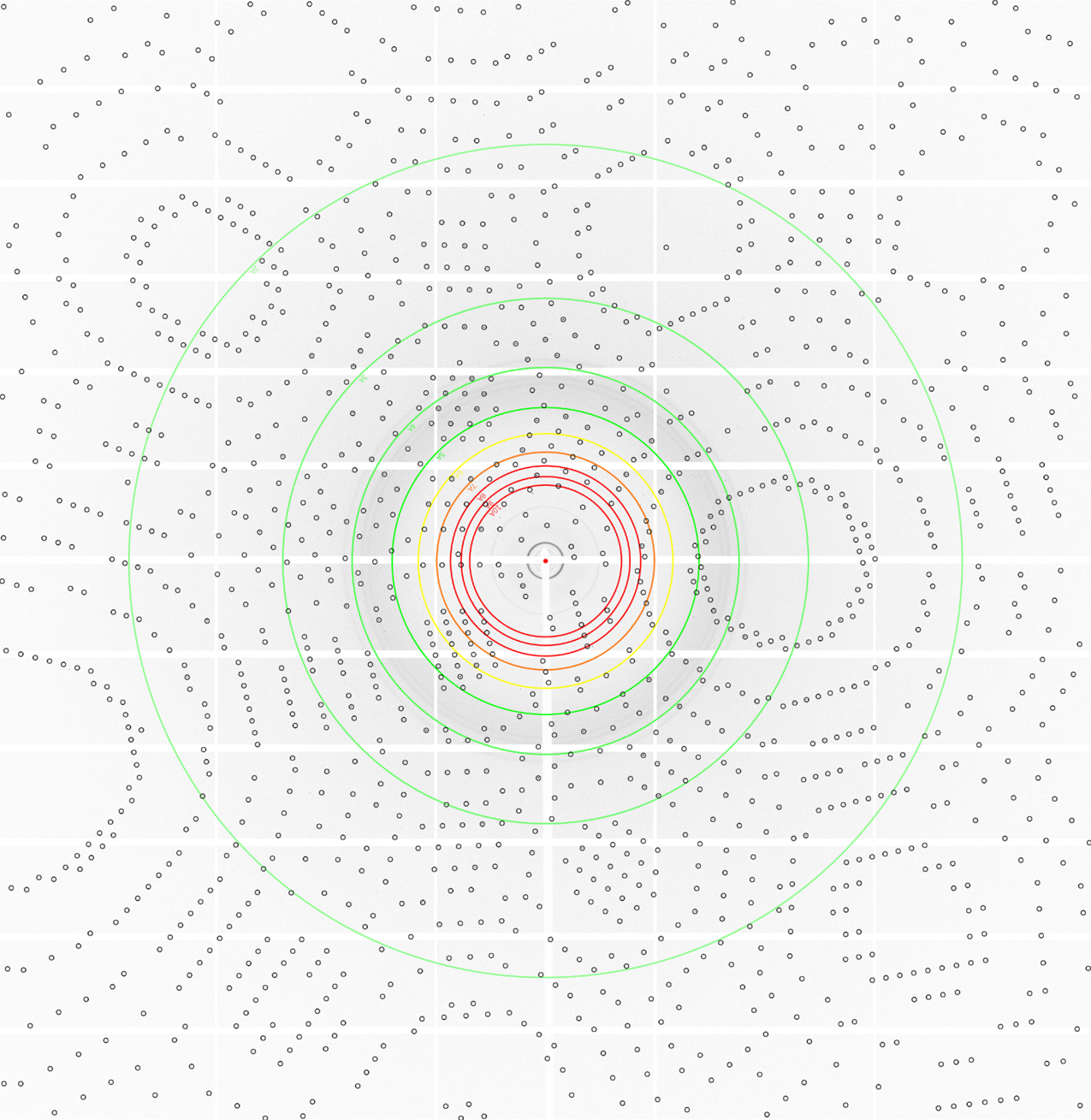


**Supplementary Figure S2.** Indexed imaged of glucose isomerase delivered in shortening A.


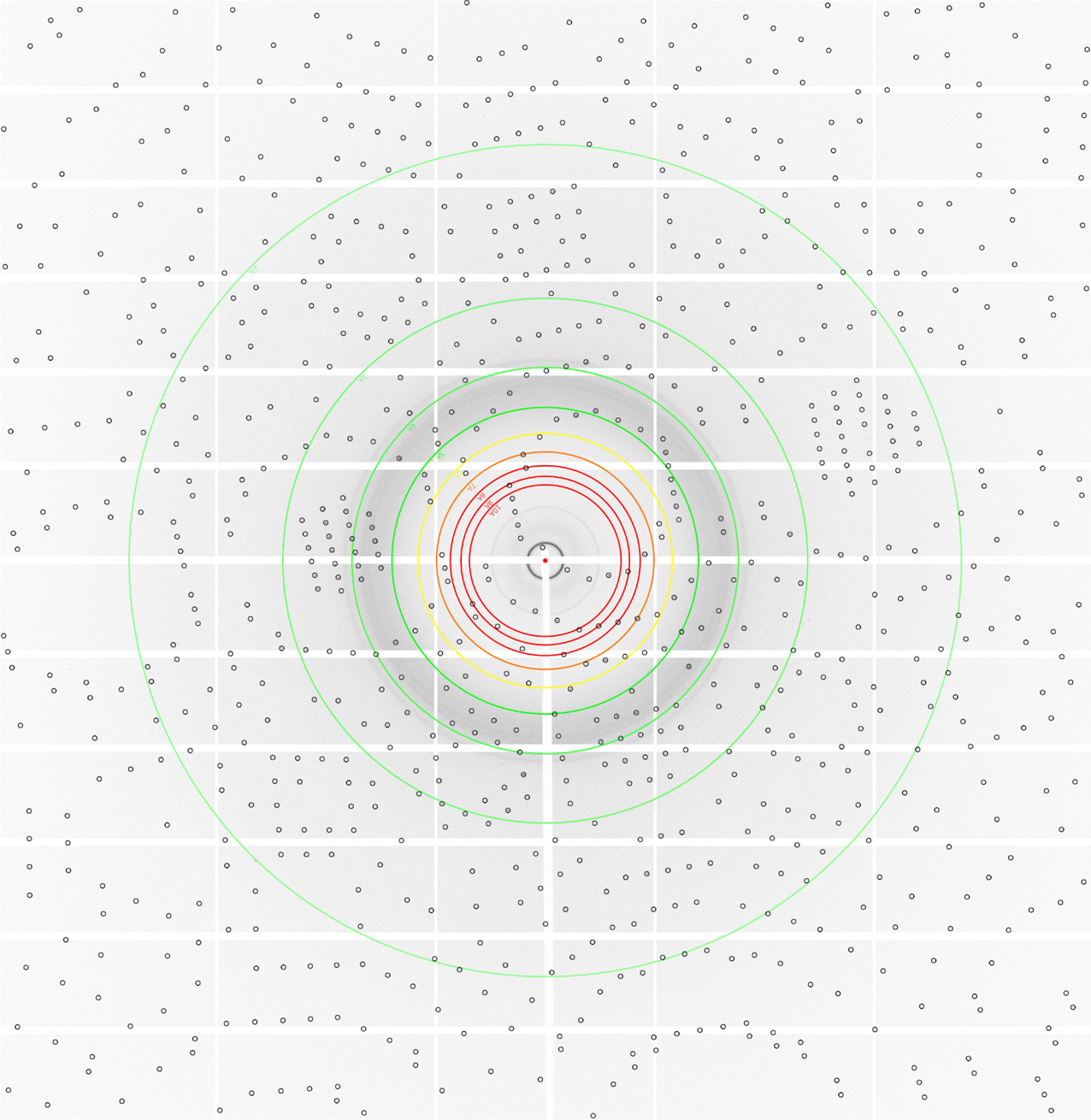
**Supplementary Figure S3.** Indexed imaged of lysozyme delivered in shortening A.


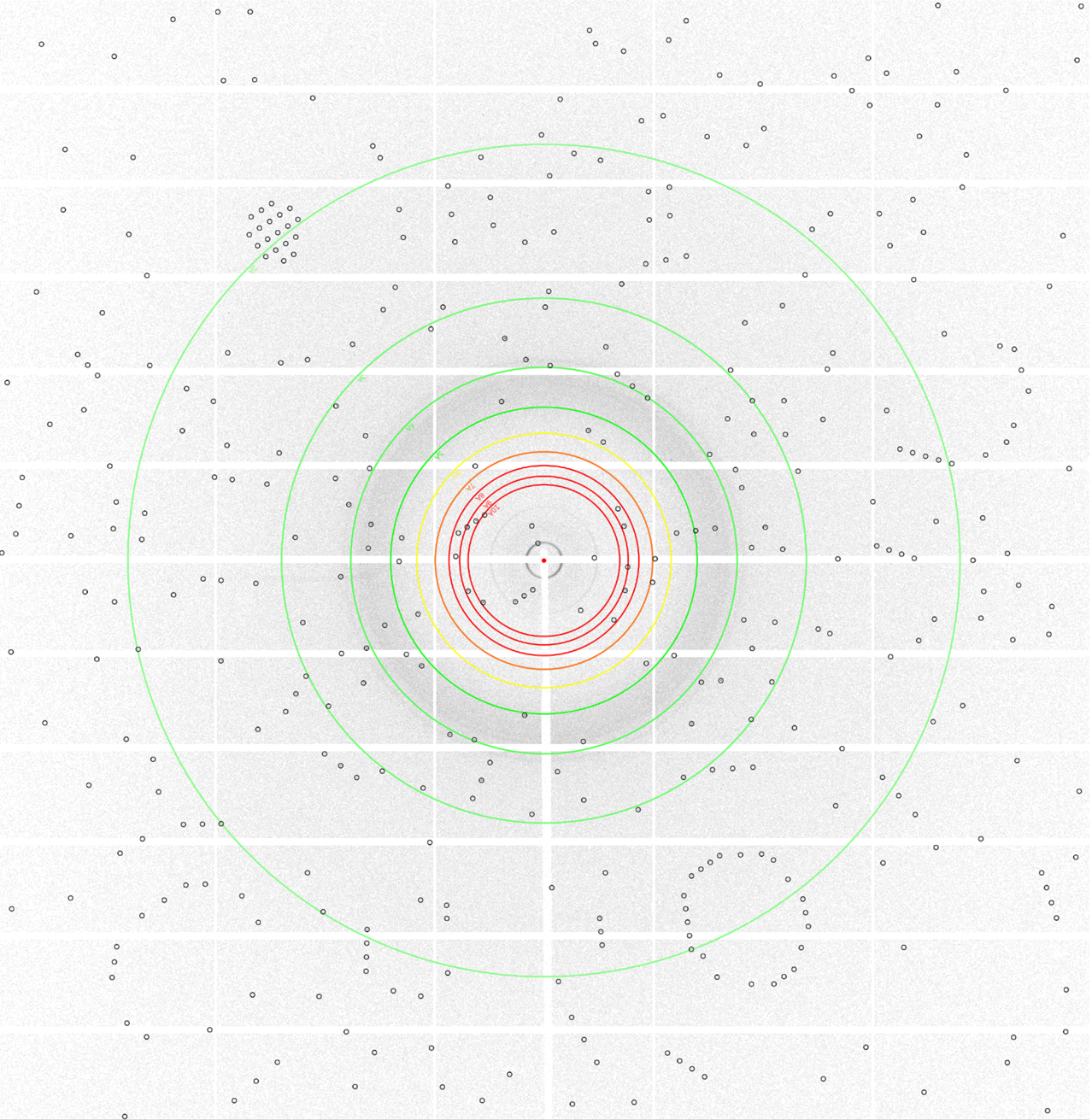


**Supplementary Figure S4.** Indexed imaged of glucose isomerase delivered in shortening B.


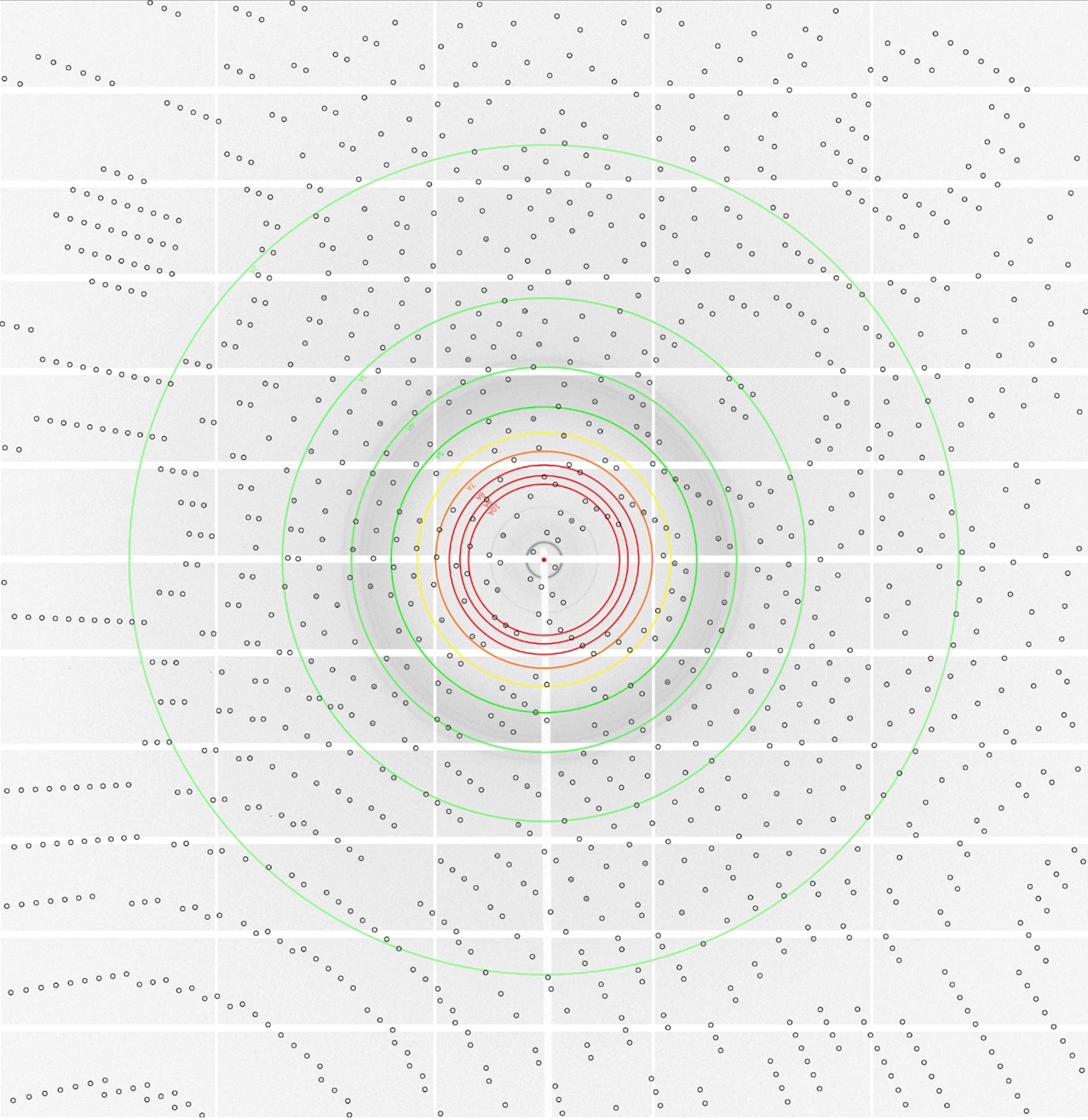


**Supplementary Figure S5.** Indexed imaged of lysozyme delivered in shortening B.


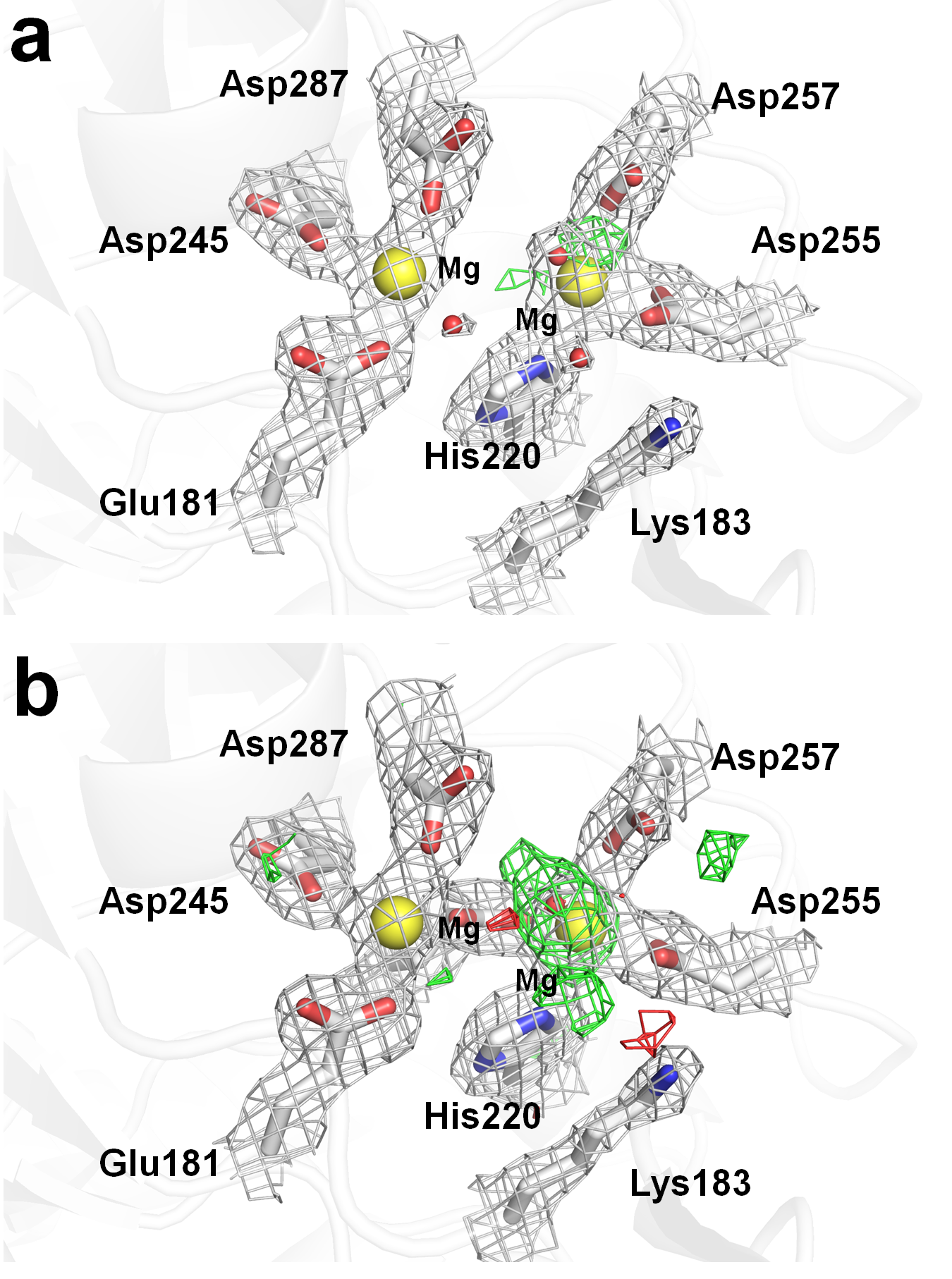


**Supplementary Figure S6.** 2Fo-Fc electron density map (grey, counted 1.5 σ) and Fo-Fc electron density map (green, counted 3 σ; red, counted - 3 σ) of active site of glucose isomerase delivered in (a) shortening A and (b) shortening B.


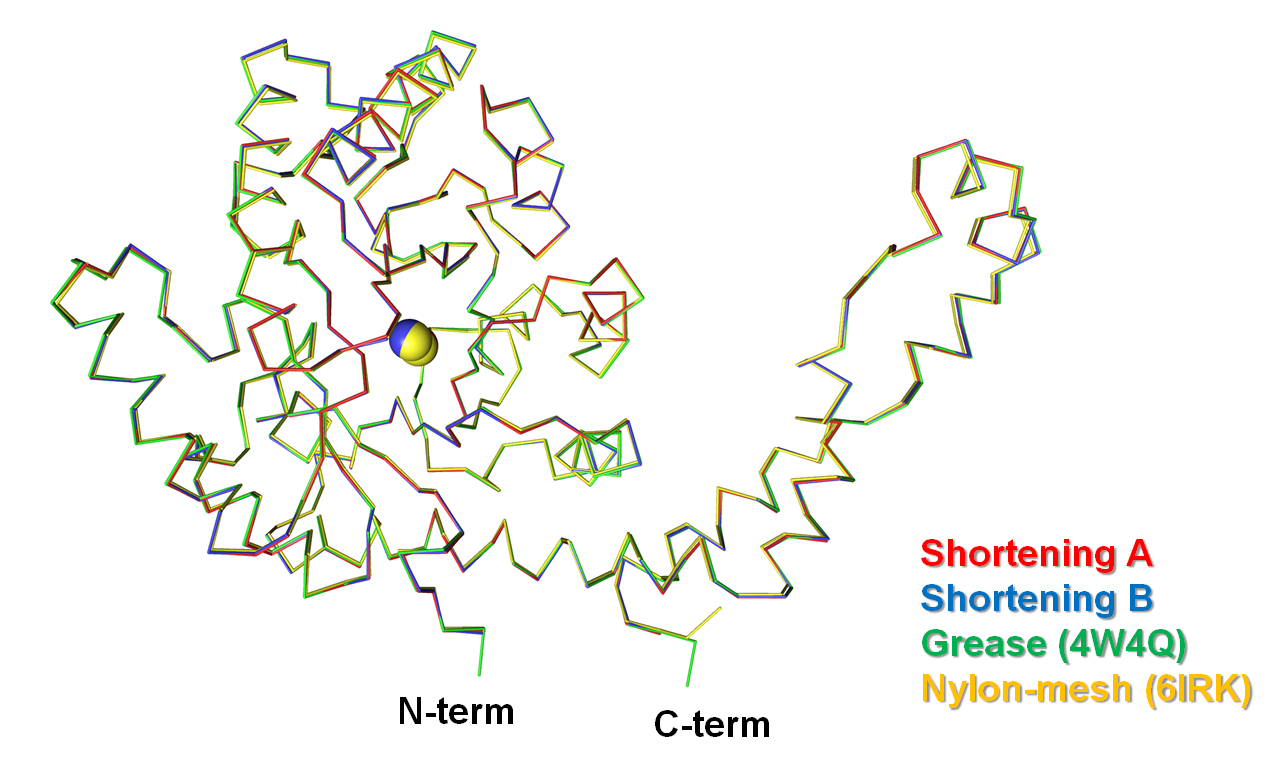


**Supplementary Figure S7.** Superimposition of the crystal structure of glucose isomerase delivered in shortening A (red) and B (blue) with glucose isomerase delivered as grease delivery medium (PDB code: 4W4Q, green) and nylon-mesh based fixed-target scanning (6IRK, yellow).


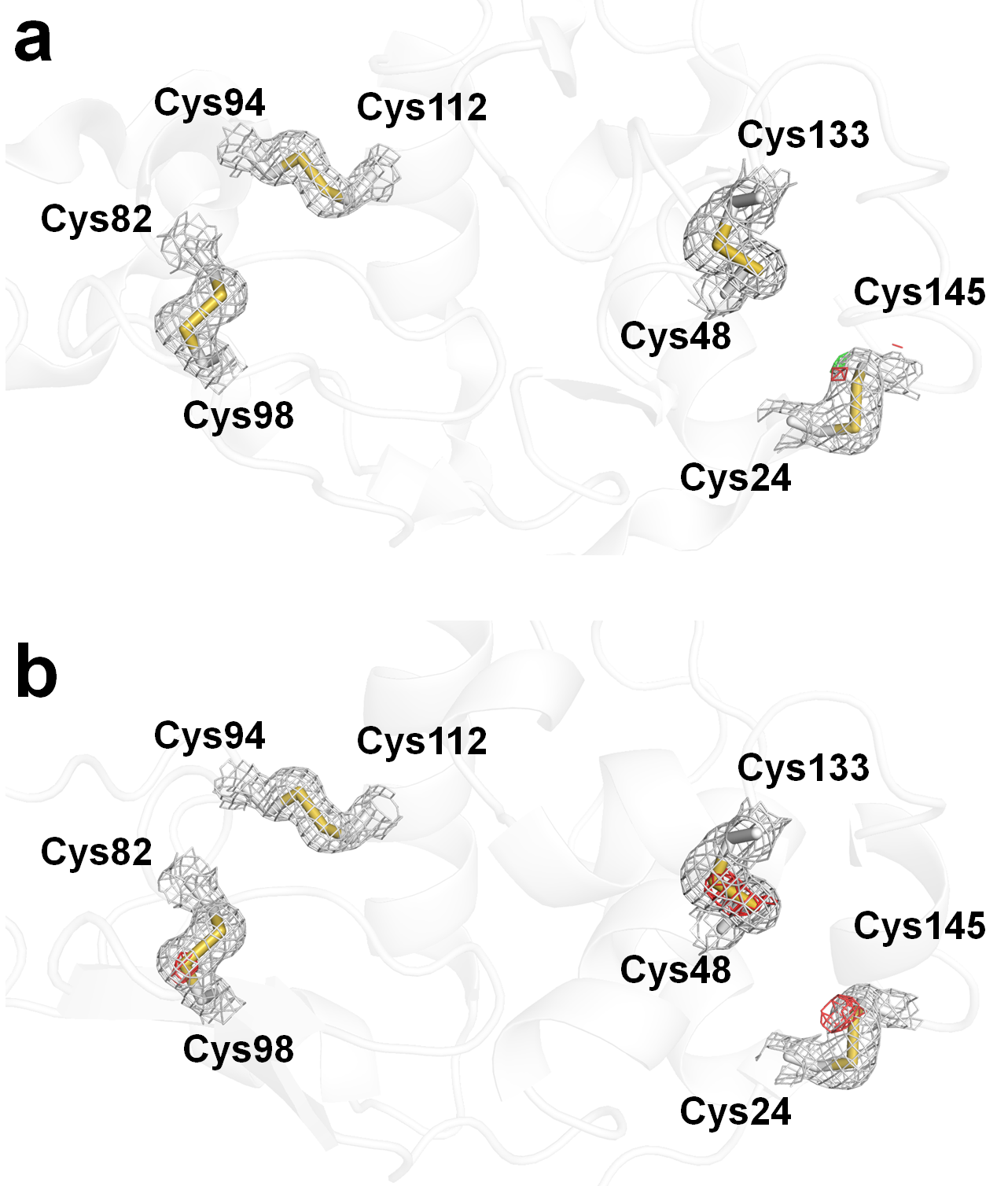


**Supplementary Figure S8.** 2Fo-Fc electron density map (grey, counted 1.5 σ) and Fo-Fc electron density map (green, counted 3 σ; red, counted - 3 σ) of disulfide bonds of lysozyme delivered in (a) shortening A and (b) shortening B.


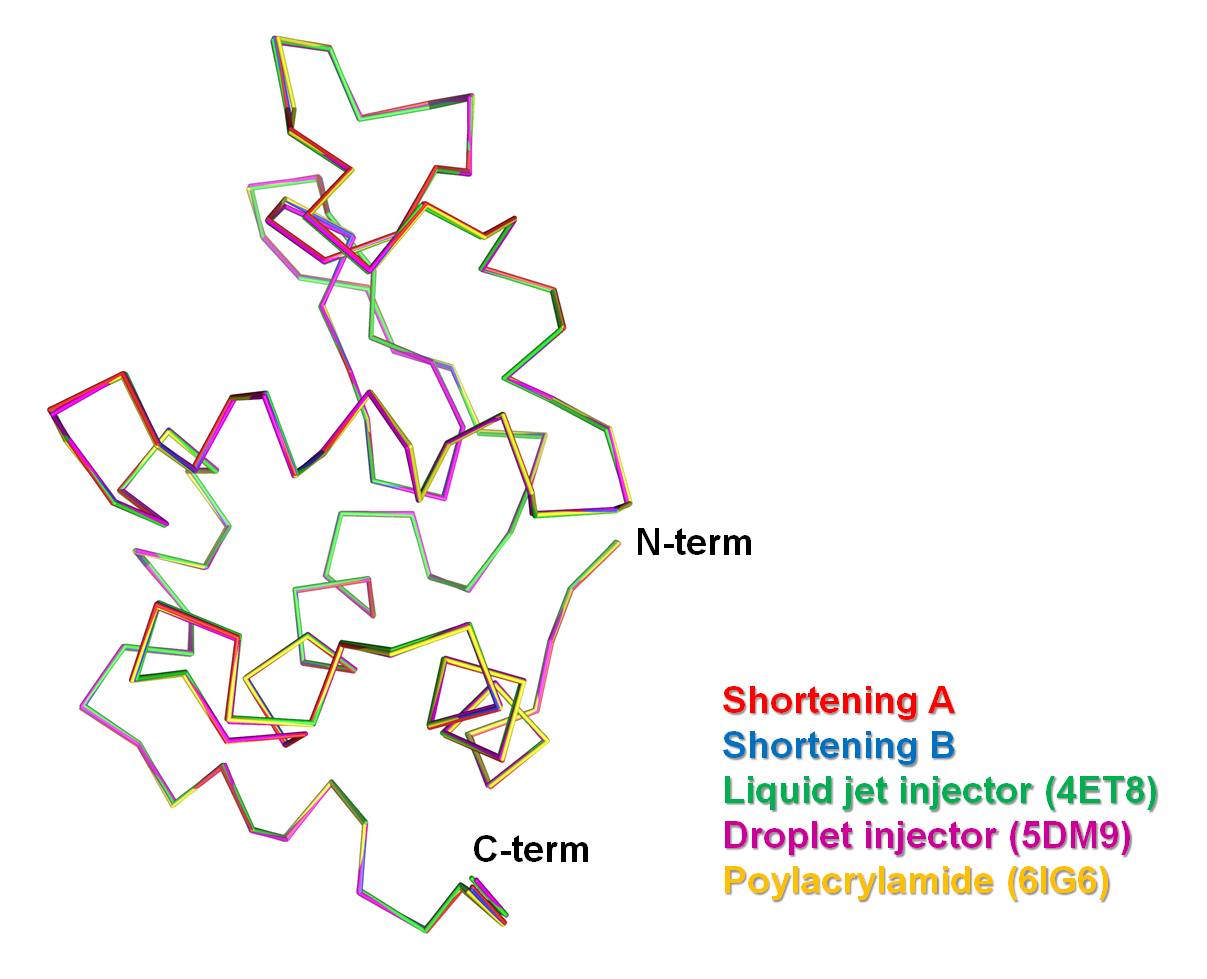


**Supplementary Figure S9.** Superimposition of the crystal structure of lysozyme delivered in shortening A (red) and B (blue) with lysozyme delivered as liquid jet with Gas Dynamic Virtual Nozzle (PDB code: 4ET8, green), droplet injector (5DM9, purple) and polyacrylamide (6IG6, yellow).

**Table S1.** Comparison of data collection statistics of lysozyme delivered in shortening A, shortening B, LCP and polyacrylamide (PAM)

|  | **Current** | | **Park and Nam ^1^** | |
| --- | --- | --- | --- | --- |
|  | **Shortening A** | **Shortening B** | **LCP** | **PAM** |
| **No. collected images** | 48000 | 48000 | 40000 | 40000 |
| **No. of hits** | 18926 | 29290 | 24258 | 24204 |
| **No. of indexed images** | 15643 | 27413 | 19411 | 21592 |
| **Resolution (Å)** | 80.00-1.80  (1.86-1.80) | 80.00-1.50  (1.55-1.50) | 78.7-1.56  (1.61-1.56) | 79.3-1.76  (1.82-1.76) |
| **Unique reflections** | 11974 (1148) | 20406 (1995) | 17418 (1685) | 12326 (1211) |
| **Completeness** | 100.0 (100.0) | 100.0 (100.0) | 99.93 (99.23) | 100.0 (100.0) |
| **Redundancy** | 1451.9 (1011.7) | 756.6 (285.8) | 318.4 (15.2) | 770.4 (526.1) |
| ***I/σ(I)*** | 7.87 (2.21) | 9.27 (1.68) | 6.00 (1.26) | 7.50 (1.60) |
| ***R*_split_** | 7.57 (46.32) | 6.58 (70.95) | 9.87 (88.66) | 7.40 (69.08) |
| **CC** | 0.9937 (0.8016) | 0.9936 (0.5183) | 0.991 (0.436) | 0.994 (0.380) |
| **CC*** | 0.9984 (0.9433) | 0.9984 (0.8262) | 0.997 (0.779) | 0.998 (0.742) |

**Reference**

1 Park, S. Y. & Nam, K. H. Sample delivery using viscous media, a syringe and a syringe pump for serial crystallography. *J Synchrotron Radiat* **26**, 1815-1819 (2019).
